# Challenges and Opportunities Associated With Platelets in Pancreatic Cancer

**DOI:** 10.3389/fonc.2022.850485

**Published:** 2022-04-12

**Authors:** Zhou Chen, Xiaodong Wei, Shi Dong, Fangfang Han, Ru He, Wence Zhou

**Affiliations:** ^1^ The First Clinical Medical College, Lanzhou University, Lanzhou, China; ^2^ Department of General Surgery, The First Hospital of Lanzhou University, Lanzhou, China; ^3^ Emergency Department, Gansu Provincial Hospital, Lanzhou, China

**Keywords:** platelet, molecular mechanism, immune evasion, targeted therapy, pancreatic cancer

## Abstract

Pancreatic cancer is one of the most common malignant tumors in the digestive system with a poor prognosis. Accordingly, better understanding of the molecular mechanisms and innovative therapies are warranted to improve the prognosis of this patient population. In addition to playing a crucial role in coagulation, platelets reportedly contribute to the growth, invasion and metastasis of various tumors, including pancreatic cancer. This narrative review brings together currently available evidence on the impact of platelets on pancreatic cancer, including the platelet-related molecular mechanisms of cancer promotion, pancreatic cancer fibrosis, immune evasion, drug resistance mechanisms, thrombosis, targeted platelet therapy, combined radiotherapy and chemotherapy treatment, platelet combined with nanotechnology treatment and potential applications of pancreatic cancer organoids. A refined understanding of the role of platelets in pancreatic cancer provides the foothold for identifying new therapeutic targets.

## 1 Introduction

Pancreatic cancer (PC) is one of the most common malignant tumors and the second leading cause of death in malignant tumors of the digestive tract (1). Importantly, it has been suggested that PC will be the second most common malignancy by 2030, given the increasing incidence in recent years (2). PC characteristics, including advanced disease stage at diagnosis and high invasion and distant metastasis rates, account for the low 1-year and 5-year survival rates (3), highlighting the need to explore novel targets to enhance the diagnostic and therapeutic approach for PC. Current evidence suggests that the average platelet count of untreated cancer patients is significantly higher than non-cancer patients or patients with prior cancer history (4, 5), suggesting that platelets play an important role in the development, progression and treatment of tumors. An increasing body of evidence suggests that thrombocytosis is related to reduced survival rate, histological type, gender, age and TNM stage for various cancers (6, 7). However, little is currently known on the molecular mechanisms and therapeutic effects of platelets in PC, warranting further investigation.

## 2 Interaction Between Various Types of Cancer and Platelets

**Figure 1:**
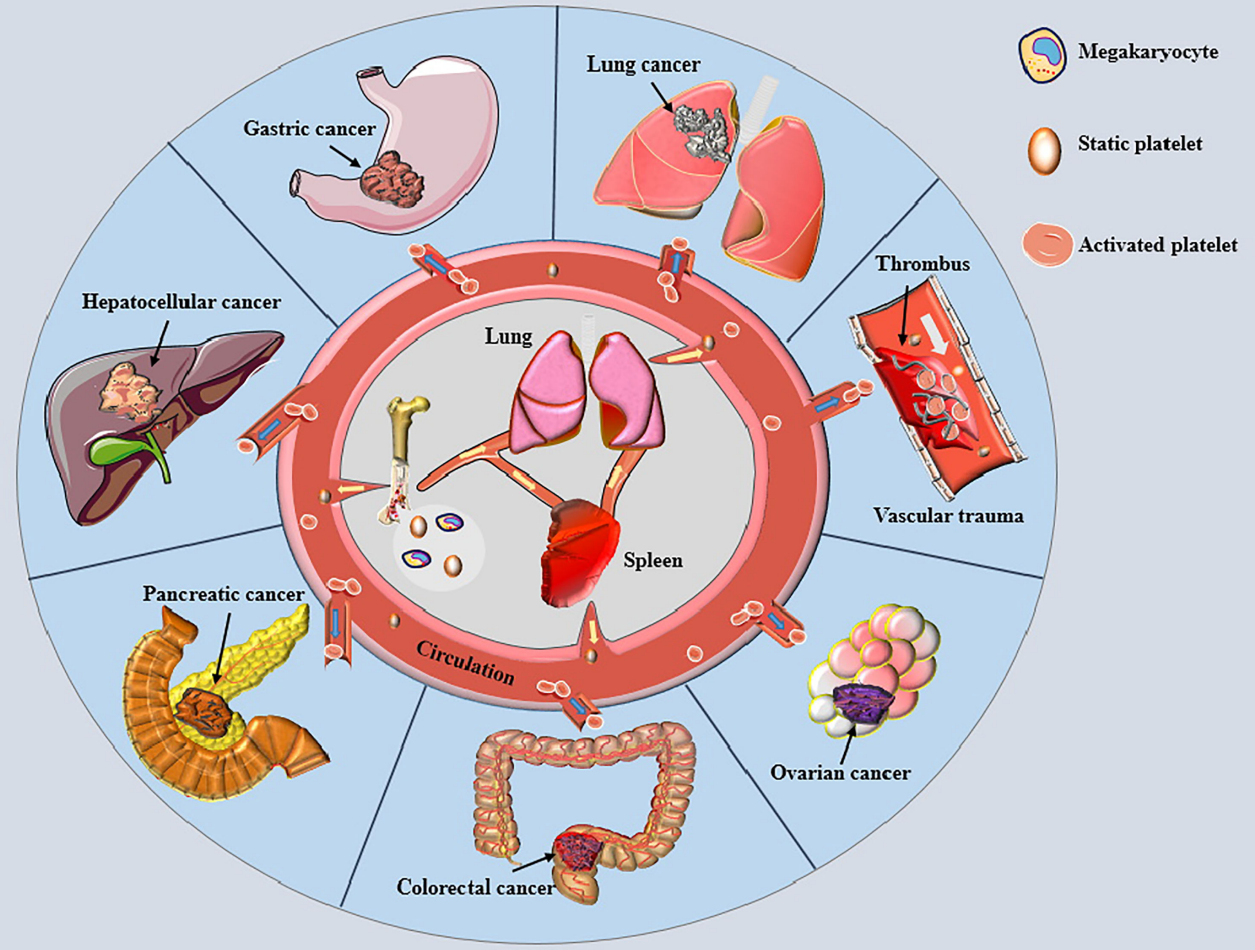
Platelets and various cancers. Small pieces of cytoplasm released from mature megakaryocyte cytoplasm in bone marrow and lung enter the circulation through the blood sinus to become static platelets. The newly generated platelets pass through the spleen, most of which are stored here and freely exchanged with the platelets in circulation to maintain the normal amount of platelets. Most of the aged platelets are removed in the spleen. When blood vessel is traumatized, platelets are rapidly activated and adhere to the wound, gathering together to form a soft hemostatic plug. Activated platelets change the malignant phenotype of tumor cells and encapsulate cancer cells to help cancer cells distant metastasis by escaping immune cell surveillance, thereby affecting the prognosises of various cancer patients.

Platelets are well-established as biologically active nonnucleated cellular fragments from the cytoplasm of mature megakaryocytes in the bone marrow, playing important roles in hemostasis and thrombosis (**Figure 1**) (8). Given that an adult body contains nearly one trillion platelets in the blood circulation and the average platelet lifespan is only about eight days, our bodies must produce 100 billion new platelets every day to keep the platelet count within the normal range (8). The newly generated platelets pass through the spleen, where one-third of the platelets are stored. The stored platelets can exchange freely with circulating platelets to maintain a normal platelet count. The spleen is also the main organ for eliminating immunocompromised platelets (9). Lefrançais et al. (10) demonstrated that massive megakaryocytes were present in the pulmonary circulation in a mouse model; megakaryocytes originating from the bone marrow and spleen sinusoids could release platelets in the lungs, accounting for 50% of the platelet count. Therefore, the lungs represent the main production site of platelets besides the bone marrow, and lung pathologies can affect the quality of platelets (11). When blood loss occurs due to vascular trauma, subcutaneous collagen or/and tissue factor (TF) is exposed to the circulation, causing quick adhesion of platelets to the wound and aggregation into clusters to form a softer hemostatic thrombus (12, 13). Under normal circumstances, the large number of circulating platelets is in dynamic equilibrium. However, platelet homeostasis is disrupted in response to different kinds of stimuli, leading to changes in platelet count and biological functions.

There is ample evidence to suggest that platelets are significantly elevated in the plasma of patients with different types of cancer (14–20). More importantly, large clinical studies showed that elevated numbers of circulating platelets were associated with tumor features, including advanced cancer and both local and distant metastasis (17, 19, 21). Meanwhile, substantial evidence suggests that thrombocytosis is predictive of poor prognosis in different cancers (22–24). Moreover, patients with higher platelet to lymphocyte ratio (PLR) were associated with shorter overall survival and disease-free survival rates (25, 26). Nevertheless, current evidence suggests poor prediction accuracy of PLR for the overall survival time of PC patients undergoing pancreatectomy (27). Thrombocytopenia induced by chemotherapeutic drugs such as gemcitabine may lead to the opposite results of the above experiments (28). In addition, increased platelet activation is a prerequisite for thrombosis. The risk of venous thromboembolism has been reported to be as high as 20% in cancer patients (29), especially in PC patients (30, 31), representing the leading cause of death in cancer patients (32). Furthermore, it has been shown that elevated platelets incredibly weaken the response and efficacy of chemotherapy drugs to tumor cells (33). In recent years, platelet inhibition combined with immunotherapy has achieved promising results for cancer treatment (34). Accordingly, the unique molecular mechanisms and advantages of platelets in tumor therapy make them potential targets for oncotherapy.

## 3 PC Cells Influence the Function of Platelets

### 3.1 PC Cells Activate and Alter the Biology of Platelets

It is widely acknowledged that platelets can be activated by various factors released by tumor cells like TF, adenosine diphosphate (ADP), thromboxane A2 (TXA2) and high-mobility group box 1 (HMGB1) (35, 36). Tumor cells can activate platelets *via* direct interactions or secretion of biologically active proteins leading to tumor cell-induced platelet aggregation (TCIPA) (37, 38). During the TCIPA process, platelet αIIbβ3, α6β1, platelet P-selectin, platelet Toll-like receptor (TLR) 4 and platelet CLEC-2 bind to protein molecules on the surface of the corresponding tumor cells, enhancing platelet activation and tumor cell malignant behavior (36, 39–42). In addition, the biological characteristics of platelets during the TCIPA process are subjected to significant changes, with tumor cell-induced platelet extracellular vesicle formation, granule release and alterations in platelet RNA profiles (37, 43). Moreover, activated platelets undergo various cellular responses, including morphological changes and translocation of membrane glycoproteins, and eventually release extracellular vesicles (EVs) containing bioactive substances (44). EVs are mainly composed of exosomes and microvesicles (MVs). Exosomes are intraluminal vesicles, 30-100nm in diameter, formed by the inward budding of endosomal membranes during maturation of multivesicular endosomes (MVEs), while MVs 100-1,000nm in diameter are generated by the outward budding and fission of the plasma membrane (45, 46). EVs induce different biological signals depending on the cell of origin. In this regard, platelet-derived exosomes originate from the extracellular secretion of multivesicular bodies and alpha granules, and MVs are produced by surface shedding (44). Furthermore, the number and proteomic profile of platelet-derived microvesicles (PMVs) exhibit variations with different stimuli (including pathologies). For instance, it has been shown that integrin α6 levels in shear stress-originated PMVs were significantly elevated compared to thrombin-induced PMVs (47). Importantly, integrin α6 has been documented in vascular endothelial growth factor-A (VEGF-A) and fibroblast growth factor-2-driven angiogenesis, promoting tumor growth *in vivo* and *in vitro* (48). Nevertheless, the proteomic alterations of platelet-derived MVs in PC remain unclear, emphasizing the need for further investigation. The effect of platelets on cancer cells may be attributed to the ability of exosomes to shuttle selected molecules, since EVs containing protein, mRNA and miRNA with biological functions can be delivered to PC cells (49). Similarly, tumor cell-derived exosomes can transfer mutated RNA to platelets by shuttling, a process that may involve plasma membrane fusion, clathrin-mediated endocytosis, and phagocytosis (50). The tumor microenvironment (TME) contains various cells, cytokines and extracellular matrix components and is the main place for the interaction between the body and the tumor. Tumor cells and platelets maintain a complex, bidirectional interaction in the TME. During TCIPA, activated platelets aggregate near tumor cells to form tumor platelet clots, protecting tumor cells from T cell immune responses and NK cell surveillance and ensuring that tumor cells can persist in circulation and metastasize to distant locations (38). Moreover, platelets contain many bioactive molecules that promote the proliferation, migration and invasion of PC cells (24, 41).

### 3.2 PC Cells Enhance Thrombopoiesis

**Figure 2:**
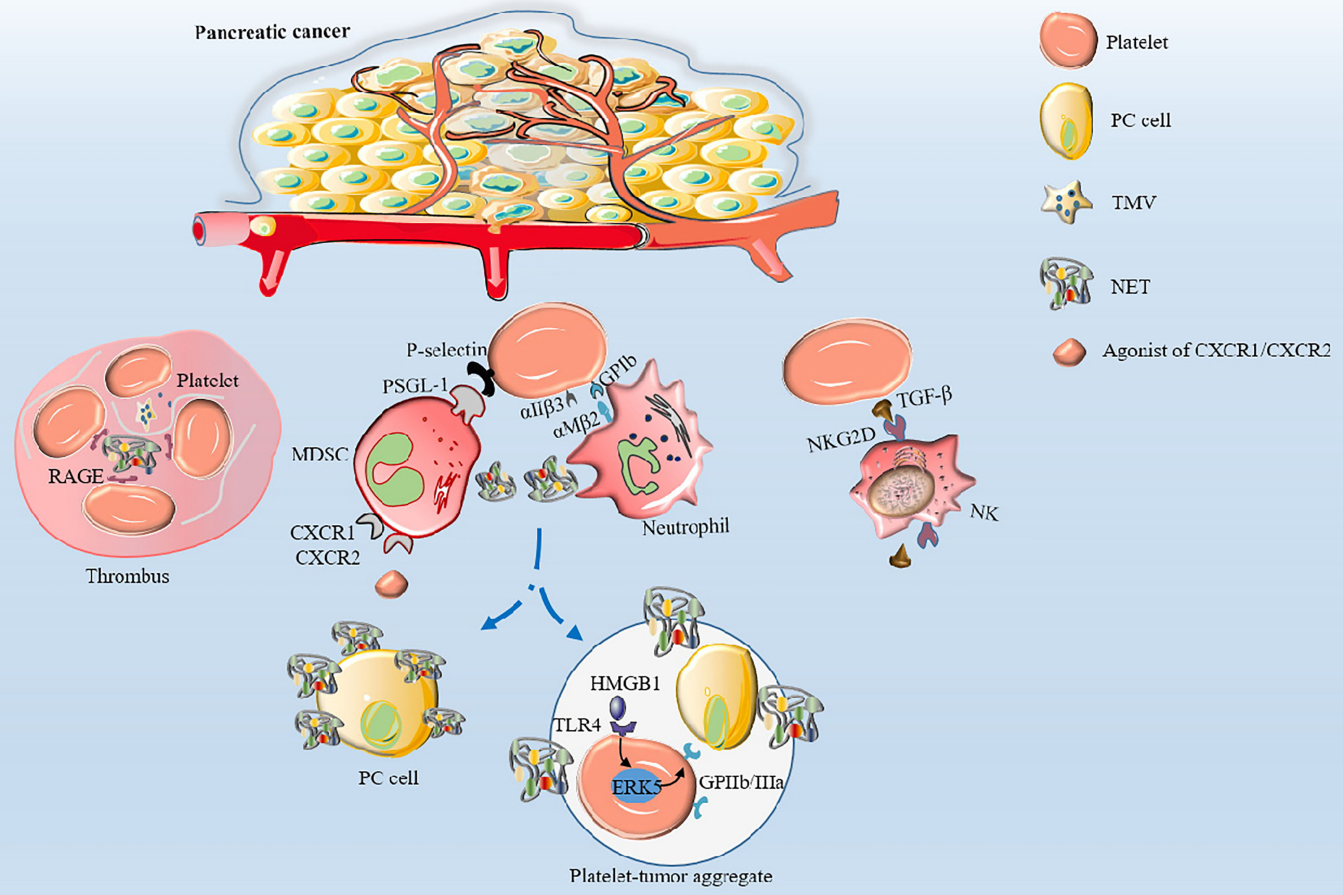
The mechanisms of thrombosis and platelet-induced evasion of immune surveillance in PC. Platelets are activated by TF and TMV incorporating large amounts of TF to aggregate to form thrombi. Activated platelets interact with integrin αMβ2 on neutrophils *via* GPIb or integrin αIIβ3 to activate and regulate the functions of neutrophils. Neutrophils release NETs, which in turn activate platelets and promote platelet aggregation *via* DNA-RAGE. Furthermore, tumor-produced CXCR1 and CXCR2 chemokine receptor agonists induce neutrophils and MSDCs to generate NETs. After surgical stress, activation of the TLR4-ERK5-integrin GPIIb/IIIa axis leads to platelet activation and formation of microaggregates with tumor cells and tumor platelet-neutrophil complexes, enhancing immune escape and leading to distant metastasis of tumor cells. Activated platelets assist PC cells to evade NK cell surveillance by releasing TGF-β to recognize NKG2D on the surface of NK cells.

It has been established that various cancer cells can activate platelets by secreting “activators” that result in venous thrombosis, the leading cause of death in cancer patients, especially in PC (30). Furthermore, studies have shown that cancer cells can secrete coagulants or fibrinolytic substances to induce platelet aggregation (51, 52). Mounting evidence suggests that membrane vesicles released by tumor cells called tumor-derived microvesicles (TMVs) incorporate large amounts of TF produced by tumor cells (52–55). TMVs are generated by outward budding and division of the plasma membrane, followed by vesicle release into the extracellular space. Using *in vitro* experiments, Geddings et al. (52) demonstrated that TMVs from human PC cells BxPc-3 and L3.6pl cells could interact with resting platelets to induce TF delivery and platelet aggregation in human and mouse plasma. In addition, after intravenous injection of TMV into mice, they found that femoral vein thrombosis and platelet deposition in the lungs was significantly increased. Using mouse models, researchers established that both TMV and TF could significantly activate platelets and increase the aggregation ability of platelets to form thrombosis by reducing the recalcification time (52, 56). In a study by Stark et al. (55), PC microvesicles (pcMVs) derived from human PC cell line L3.6pl were injected into mice by intravenous injection. It was found that pcMVs could selectively promote thrombus growth in regions with slow and turbulent flow and significantly shortened whole blood clotting time, leading to the formation of large thrombi upstream of the stenosis. Furthermore, activated platelets can directly activate neutrophils and induce the formation of neutrophil extracellular traps (NETs) (57). Conversely, NETs can induce thrombin production and activate platelets to release ATP and ADP, causing a cascade of reactions and promoting platelet aggregation (58, 59). It has been established that during NETs formation, DNA, TF, myeloperoxidase and histones are released, and DNA upregulates platelet aggregation through the platelet receptor for advanced glycation end products (RAGE) (60). Thrombosis is showed in **Figure 2**. However, NETs were not observed in the thrombi in a study where a minimally invasive laser-induced injury model was used, although neutrophils were present at the injury site (61). Interestingly, activated platelets gather into clusters and form thrombi in the TME; however, the thrombi are different from those caused by benign diseases. Histological analyses confirmed that pcMV-thrombi had a composition distinct from nonmalignant thrombi. In contrast, a study reported that the luminal area in the pancreatic TMV-thrombi was filled with a loose fibrin (-proto) network, while neutrophils, monocytes and platelets were significantly reduced (55). Portal vein thrombosis is well-recognized as the most common type of thrombosis in patients with advanced PDAC, followed by mesenteric vein thrombosis and splenic vein thrombosis, suggesting its value as an indicator of poor prognosis (62, 63). Nevertheless, it has been reported that thrombocytosis has little to do with the increased incidence of thromboembolism (6) since the hypercoagulable state of the whole body is not related to an absolute increase in specific factors but the presence of activated coagulation factors (64). Overall, high expression levels of TMV, NET and TF in PC tissue participate in platelet activation and aggregation, which coupled with procoagulant molecules enzyme heparanase (HPSE), podoplanin (PDPN) and the fibrinolytic system, contribute to the high incidence of thrombosis, making it an excellent model for studying cancer-associated hypercoagulable states (65).

## 4 Activated Platelets Provide Favorable Environment for PC Cells

### 4.1 Activated Platelets Alter the Malignant Phenotype of PC Cells

**Figure 3:**
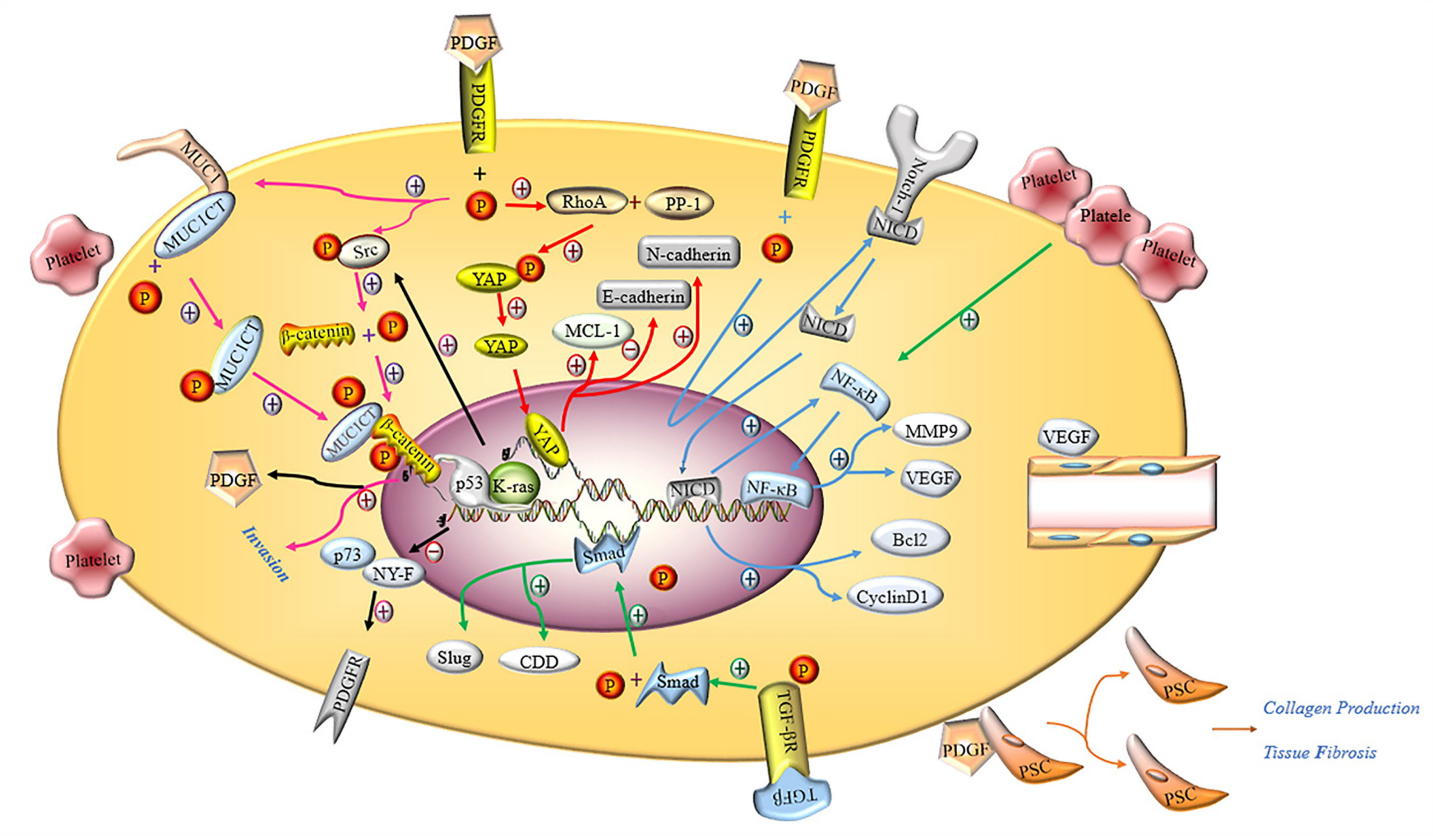
Molecular mechanism of activated platelets inducing malignant phenotype of PC cells. PDGFR activated by PDGF induces the phosphorylation of tyrosine residue in the intracellular domain, activating the Hippo/Yes-associated protein signaling pathway and promoting MCL-1, N-cadherin and inhibiting E-cadherin (red arrow). Notch-1 is promoted by activated PDGFR and releases intracellular domain of Notch 1 (NICD), which enters the nucleus and increases the expression of Bcl2, Cyclin D1 and NF-κB. NF-κB promotes the expression of MMP9 and VEGF by binding nuclear genes (blue arrow). Activated PDGFR induces phosphorylation of MUC1CT and Src, phosphorylation of Src induces phosphorylation of β-catenin, and the combination of phosphorylated MUC1CT and β-catenin enhances the invasion of PC cells (pink arrow). PDGF induces PSC to proliferate and secrete collagen, thereby aggravating pancreatic fibrosis. The deletion of p53 and the mutation of K-ras not only inhibits the binding of p73 and NY-F, so that the activation of PDGFR is not inhibited, but also induces β-catenin phosphorylation through Src phosphorylation to promote the expression of PDGF (black arrow). Activated TGF-βR promotes the expression of Smad, and the Smad protein enters the nucleus to promote the expression of CDD and Slug (green arrow). VEGF induces the proliferation and remodeling of endothelial cells in the TME.

Ponert et al. (66) demonstrated that different cancer cells could easily bind to activated platelets *in vitro*, thereby accelerating the adhesion between platelets and tumor cells; this phenomenon was particularly prominent in PC cells. In another *in vitro* experiment, investigators observed that after many platelets aggregated by binding to PC PANC-1 cells, the migration, invasion and proliferation capacity of PANC-1 cells was significantly enhanced (24, 67). The cytoplasm of platelets contains many biologically active proteins, such as growth factors, chemokines, cytokines and proteases, which are secreted by activated platelets (68). The main documented molecular pathways are shown in **Figure 3**.

#### 4.1.1 Roles of Platelet-Derived Growth Factor

Platelet-derived growth factor (PDGF) plays an important role in maintaining the integrity of blood vessels in the TME, promoting the proliferation of tumor cells, epithelial-mesenchymal transition (EMT) progression and tumor metastasis in PC (69). According to the literature, PDGF-BB exerts no effects on the proliferation of tumor cells but enhances the invasion and metastasis of tumor cells through matrigel *in vivo*, resulting in PDGFR-β-mediated phosphorylation of MUC1 cytoplasmic tail (MUC1CT) to regulate the invasiveness of PC cells. MUC1 is a type I transmembrane protein that is overexpressed and abnormally glycosylated in ductal adenocarcinoma (70). Besides, another study revealed that autocrine PDGF-BB significantly increased the proliferation, migration and invasion of PC cells *via* the Hippo/Yes-associated protein signaling pathway (71). Metalloproteinase-9 (MMP-9) belongs to the matrix metalloprotein family, whose main function is to degrade and remodel the dynamic balance of the extracellular matrix. Current evidence suggests that MMP-9 is related to tumor pathological features, including invasion, metastasis, and angiogenesis (72). Suzuki et al. (7) reported that the invasive ability of PC cells co-cultured with platelets was significantly enhanced as platelets stimulated PC cells to secrete more MMP-9. It is well-recognized that Notch-1 signaling plays an important role in maintaining the balance between cell proliferation, differentiation and apoptosis (73). A study by Wang et al. (74) illustrated that downregulation of PDGF-D effectively limited the invasive ability of PC cells through inactivation of Notch-1 and NF-κB DNA binding activity, which in turn downregulated the expression of their target genes VEGF and MMP-9. In contrast, the opposite results were observed with overexpression of PDGF-D by cDNA transfection. In addition, the conditioned medium from cells transfected with PDGF-D siRNA showed significantly reduced levels of vascular endothelial growth factor (VEGF), which in turn inhibited tube formation by human umbilical cord vascular endothelial cells.

Studies have shown that the ligand PDGFR is indispensable for PDGF to maximize its biological function. Researchers discovered that β-catenin activation, coupled with K-ras mutation and loss of p53, could activate the autocrine PDGF/Src signal and significantly increase the proliferation and distant metastasis of PC cells, accounting for the poor prognosis of PC (75). Notably, missense mutations in the p53 tumor suppressor play an indispensable role in tumor proliferation, invasion, migration and metastasis. PDGFR is a downstream mediator of mutant p53 that has been reported to harbor huge potential for maintaining the aggressiveness of PC cells by disrupting the formation of the p73/NF-Y, a complex whose interaction prevents it from binding and activating the PDGFR promoter (76).

#### 4.1.2 Roles of Vascular Endothelial Growth Factor

VEGF is a highly specific vascular endothelial cell growth factor that increases vascular permeability and promotes the degeneration of extracellular matrix, migration and proliferation of vascular endothelial cells and blood vessel formation (77, 78). It has been shown that VEGF is abundantly stored in platelets at higher concentrations than in plasma and is related to the poor survival rate of PC patients (77, 79). Importantly, VEGF effectively promotes the proliferation, invasion and metastasis of tumor cells, as well as angiogenesis in the TME (80, 81). Besides, VEGF overexpression-induced tumor microangiogenesis is closely related to the microvessel density (MVD) in PC tissues, promoting local tumor growth by paracrine signal transduction in stromal cells expressing VEGFR and allowing cancer cells to invade peritumoral lymphatic vessels (77). Mesenchymal stem cells (MSC) in the TME can secrete VEGF, contributing to angiogenesis in PC (82). It has been reported that PC cells express the functional P2Y12 receptor required for cell proliferation by promoting EGFR-dependent and independent AKT-mediated survival signals (83). In an *in vitro* model of angiogenesis, Battinelli et al. (84) observed that activated platelets significantly released more VEGF and promoted the formation and migration of human umbilical vein endothelial cells capillary structure, and enhanced tumor growth.

#### 4.1.3 Roles of Transforming Growth Factor-β1

Transforming growth factor-β1 (TGF-β1) belongs to the newly discovered TGF-β superfamily that regulates the growth and differentiation of cells. Slug is a transcription-related factor of the EMT that has been reported to be mainly regulated by TGF-β1 through the Smad effector pathway (85). Current evidence suggests that Slug expression is significantly increased in PC cells exposed to platelet releasate (PR)-TGF-β1 and can induce EMT progression. Platelet-derived TGF-β and direct platelet-tumor cell contraction can synergistically activate the TGF-β/Smad and NF-κB pathways in cancer cells, exhibiting an aggressive mesenchymal phenotype and enhanced metastasis *in vivo* (85). In addition, studies have shown that TGF-β1 in the TME could effectively induce pancreatic stellate cells (PSCs) to secrete alpha-smooth muscle actin (αSMA), thereby exacerbating fibrosis while inhibiting the toxic response of CD8^+^ T cells to PC cells (86).

#### 4.1.4 Roles of MiRNAs

MiRNAs are widely acknowledged to bind to specific regions of target gene mRNAs, which degrade or inhibit mRNAs and subsequently lead to inhibition of protein translation (87, 88). Serious platelet-related diseases are caused by dysfunctions of the miRNA-based regulatory system (89); the regulatory mechanism is controlled by platelet-specific signals and is not restricted by the nucleus (90). Studies have shown significant differences in the miRNA expression profiles in the blood circulation of PC patients and patients with benign pancreatic diseases (87). Recent research has demonstrated that miR-221-5p, miR-29a-3p, miR-22-3p and miR-17-3p were abundant in platelets of PC patients, and miR-29a-3p could inhibit the expression of SPARC, a multifunctional glycoprotein, and promoted proliferation, migration and invasion of PC cells in vitro (91). In addition, miR-221 has been reported to be essential for PDGF-mediated EMT phenotype, migration, and growth of PC cells (92). Another study showed that depletion of miRNA-rich platelets led to a marked increase in the growth rate of PC; however, the specific miRNAs and target genes have not been identified (93). Overall, platelets contain many unknown non-coding RNAs, including miRNAs, which potentially participate in the pathogenesis of PC.

#### 4.1.5 Roles of Other Protein Factors

An increasing body of evidence suggests that ADP derived from ATP released from pancreatic acinar cells and dense granules of platelets (35, 94) can effectively stimulate and activate platelets *via* Gq-coupled P2Y1 and Gi-coupled P2Y12 receptors located on the platelet membrane (95). The surrounding platelets are activated by ADP and trigger a cascade reaction that activates more platelets releasing VEGF and promoting tumor proliferation (84). It has been shown that platelet glycoprotein (GP) is involved in platelet adhesion, aggregation and activation and mediates the combination of platelets and CD34^+^ cells from human blood and bone marrow *via* P-selectin (96). Increased CD34 expression has been established to promote the invasion and migration of PDAC cells (97). Nevertheless, the role of membrane glycoproteins of human platelets in PC is still unclear. Platelet thrombospondin-1 (TSP-1) is a platelet alpha-granule and matrix glycoprotein involved in tumor invasion, angiogenesis and metastasis. TSP-1 has been established as a regulator of angiogenesis that is strongly expressed in PCs, upregulates the production of MMP-9 and contributes to the extensive neovascularization and spread of highly aggressive tumors (98, 99). Boone et al. (100) demonstrated that the nucleotide-binding domain leucine-rich repeat-containing protein 3 (NLRP3) in platelets was upregulated in mice models and led to significant platelet aggregation *in vivo*. Importantly, NLRP3 forms a complex with the adaptor protein apoptosis-associated speck-like protein containing a caspase activation and recruitment domain (ASD) to promote PC progression. The opposite results were observed with NLRP3 inhibitors, with inhibited growth of PC cells and improved survival rate of mice. Moreover, P-selectin accelerates thrombus formation, induces infiltration of MDSCs and evades immune cytotoxic effect *via* PSGL-1 (101, 102). Platelet factor 4 (PF-4) reportedly regulates the activity of fibroblast growth factor 2 (FGF-2), resulting in the phosphorylation of E-cadherin and β-catenin on tyrosine residues leading to angiogenesis (103, 104). Activation of EGFR by epidermal growth factor (EGF) induces phosphorylation of PLCγ, which ultimately leads to high spontaneous migratory activity in PC cells (105). Moreover, platelet-derived lysophosphatidic acid (LPA) enhances the invasion and migration of PC cells through LPAR (106). Last but not least, the Von Willebrand factor (VWF) can activate platelets to promote platelet aggregation and emboli formation *via* GPIb, promoting tumor metastasis (107, 108).

### 4.2 Activated Platelets Enhance Drug Resistance of PC Cells

The enzyme cytidine deaminase (CDD) has been reported to participate in the mechanism of gemcitabine resistance by intracellular metabolism of gemcitabine (109) after its upregulation by platelet releasate (110). Human ENT1 (hENT1) is well-known for enhancing the cellular uptake of gemcitabine, thereby enhancing its toxic effects in PC cells (111). Moreover, Slug is a master regulator of EMT that is highly expressed in CD133^+^ human PC cell lines (Capan-1) and enhances the migration and invasion of PC cells, resulting in gemcitabine resistance (112). *In vitro* experiments have shown that platelet-derived ADP and ATP induced high Slug expression *via* P2Y1 and P2X7 receptors on the surface of human PC cell lines AsPC-1 and BxPC-3. Importantly, a study demonstrated that Slug could effectively inhibit hENT1 expression, stimulate CDD expression, and enhance the resistance of PC cells to gemcitabine by inhibiting the uptake of gemcitabine by PC cells and accelerating the metabolism of gemcitabine (35, 113). Activated platelet-derived TGF-β1 stimulates PI3K/Akt and MEK/Erk signaling in PC cells, resulting in decreased cisplatin sensitivity (114). Nonetheless, the regulatory role of platelets on the efficacy of PC chemotherapy remains unclear, warranting further study. The above findings suggest that activated platelets can mediate drug resistance in PC cells to a certain extent.

### 4.3 Activated Platelets Contribute to PC Fibrosis

The varying degrees of fibrosis associated with PC account for the difficulty of providing effective treatment for this patient population. Tumor cells are hidden in a thick fibrotic matrix that acts as a barrier and is responsible for the poor response to chemotherapy drugs. Over the years, research on the mechanism of platelet fibrosis in PC has been limited to PSCs. PSC has been acknowledged to play a key role during pancreatic fibrosis in chronic pancreatitis and the pro-fibrotic reaction of PC by producing the stromal reaction. Studies have shown that activated platelets could effectively activate PSC and promote the formation of connective tissue (75). In a mouse model experiment, Vonlaufen et al. (115) demonstrated that the PC group co-cultured with PSC exhibited a faster growth rate, larger volume and more fibrotic bands containing activated PSC. Moreover, PSC migration was significantly increased by PC cells *in vitro*. On the contrary, the secretion of PSC could induce PC cell proliferation and migration and inhibit apoptosis. PDGF and TGF-β released by activated platelets have been recognized as effective stimulators for PSC proliferation to accelerate extracellular matrix synthesis (115). Fitzner et al. (116) elucidated that the activation of rat PSCs *in vitro* was related to increased expression of galectin-1, and galectin-1 could mediate PSC function. PDGF stimulated the expression of the lectin galectin-1 resulting in high proliferation rates and synthesis of more collagen. Targeting platelets against PC fibrosis is a potential therapeutic approach.

### 4.4 Activated Platelets Assist PC Cells to Evade Immune Surveillance

When tumors and associated blood vessels are destroyed, cancer cells escape and slough off into the circulation to form circulating tumor cells (CTCs), which become the seeds for distant metastasis of tumors (117). Interestingly, platelets can couple to tumor cells, increase vascular permeability and induce extravasation of tumor cells (118). Tumor cells entering the circulation must deal with high shear rates and immune surveillance, such as NK cell attacks. Eventually, only a small proportion of tumor cells enter the blood circulation for metastasis, making this process very inefficient (119). NK cells play important roles in cancer immune surveillance by mediating direct cytotoxicity and releasing immunomodulatory cytokines to form an adaptive immune response and prevent tumor progression and metastasis. During hematogenous metastasis, cancer cells are quickly encapsulated by platelets, similar to cancer cells putting on a “protective suit”, making it impossible for NK cells to recognize tumor cells allowing distant metastasis (**Figure 2**). Interestingly, researchers found that thrombocytopenia could effectively inhibit the ability of cancer cells to metastasize in mice models. This phenomenon was reversed by the depletion of NK cells and secretion of TGF-β by activated platelets, thereby inhibiting immunoreceptor natural killer group 2, member D (NKG2D), indispensable for antitumor activity of NK cells (120). Okazaki et al. (121) noted that platelets could preferentially adhere to mesenchymal cells rather than epithelial cells in the TME of mouse models of infectious disease. Interestingly, cancer cells could be wrapped by activated platelets to escape immune surveillance and promote metastasis.

Neutrophils are important innate immune cells in the blood circulation that play important roles in innate and adaptive immunity. Activated platelets can recruit neutrophils by releasing chemical mediators such as CXCL4 (122) and directly interact with integrin αMβ2 on neutrophils *via* GPIb or integrin αIIβ3 to activate and regulate the functions of neutrophils (123, 124). After activation by various stimuli (e.g., infection, surgery, activated platelets), neutrophils can release reticular ultrastructures composed of protein-studded chromatin called NETs (52, 125, 126). NETs play a double-edged role. On the one hand, they play a positive role in the invasion of pathogenic microorganisms. On the other hand, NET amplifies platelet activation, aggregation and thrombin activation, promotes intravascular coagulation, and promotes the attachment of cancer cells to the blood vessel wall, resulting in enhanced tumor migration (127). In this regard, it has been reported that after surgical stress, activation of the TLR4-ERK5-integrin GPIIb/IIIa axis leads to platelet activation and formation of microaggregates with tumor cells and tumor platelet-neutrophil complexes, enhancing immune escape and leading to distant metastasis of tumor cells (128). Importantly, thrombomodulin effectively prevents PC metastasis to the liver by degrading HMGB1 and thus inhibiting the induction of NETs (129). Furthermore, tumor-produced CXCR1 and CXCR2 chemokine receptor agonists induce neutrophils and granulocyte myeloid-derived suppressor cells (MDSCs) to generate NETs that encapsulate tumor cells and protect them from the cytotoxicity of CD8^+^ T cells and NK cells by hindering the contact between immune cells and surrounding target cells (130). NETs can suppress T-cell responses through metabolic and functional exhaustion, promoting tumor growth (131). However, the role of activated platelets in evasion of immune surveillance and immune cytotoxicity by PC cells remains to be elucidated. The studies of potential markers associated with platelets in PC are summarized in **Table 1**.

**Table 1:** Studies on potential markers associated with platelets in PC.

Potential marker	Mechanism of action	Effect on tumor	*In vitro*/*in vivo*	References
PDGF	PDGF-PDGFR-MUC1CT; PDGF-PDGFR-YAP-MCL-1/N-cadherin; PDGF-PDGFR-Notch-1 and NF-κB-VEGF/MMP-9	Enhanced the invasion and metastasis of tumor cells; Promoted angiogenesis	Both	(70, 71, 74)
VEGF	PDGF-Notch-1 and NF-κB-VEGF	Increases vascular permeability; Promoted the migration and proliferation of vascular endothelial cells and blood vessel formation	Both	(74, 78)
TGF-β1	TGF-β-Smad and NF-κB; TGF-β1-PI3K/Akt and MEK/Erk	Enhanced EMT and drug resistance	Both	(85, 114)
ADP/ATP	ADP-P2Y1R/P2Y12R-VEGF; ADP/ATP-P2Y1/P2X7R-CDD	Amplified platelet degranulation and aggregation; Enhanced drug resistance	Both	(95, 113)
GP	GP-P-selectin-CD34	Promoted activated platelet adhesion to PC cells and tumor proliferation	*In vitro*	(96)
TSP-1	TSP-1-MMP-9	Promoted tumor invasion, angiogenesis and metastasis	*In vitro*	(98, 99)
NLRP3	NLRP3-ASC	Promoted activity and aggregation of platelets, and PC cell progression.	*In vivo*	(100)
P-selectin	P-selectin-PSGL-1	Accelerated thrombus formation; Induced MDSCs infiltration; Evaded immune cytotoxic effect	Both	(101, 102)
EGF	EGF-EGFR-PLCγ	Enhanced the migration of PC cell	*In vitro*	(105)
VWF	VWF- GPIb	Promoted platelet aggregation and emboli formation	Both	(107, 108)
α6β1	α6β1-ADAM9	Enhanced tumor metastasis	Both	(40)
αIIbβ3	αIIbβ3-PI3K-c-MYC	Induced PC cell proliferation	*In vitro*	(67)
PF-4	PF-4-FGF-2-E-cadherin/β-catenin	Increased neovascularization	*In vitro*	(103, 104)
LPA	LPA-LPAR	Enhanced PC cell invasion and migration	*In vitro*	
miR-29a-3p	miR-29a-3p-SPARC	Promoted proliferation, migration and invasion of PC cells	*In vitro*	(91)
miR-221	miR-221-PDGF	Mediated EMT phenotype, migration and proliferation of PC cells	*In vitro*	(92)

## 5 Applications of Platelets in the Treatment of PC

### 5.1 Platelet-Related Targeted Therapy in PC

At present, the application of platelets to enhance antitumor therapeutic effects is widely used in preclinical studies and clinical trials of PC, emphasizing the inhibition of platelet activation and abnormal pathways of cancer cells associated with activated platelets. The efficacies of single antiplatelet drugs and a combination of antiplatelet and chemotherapy drugs have been assessed in these studies. Aspirin is widely acknowledged as a derivative of salicylic acid with antiplatelet properties. It has a significant inhibitory effect on platelet aggregation and effectively prevents thrombosis by inhibiting the production of cyclooxygenase. Accordingly, it is widely used in clinical practice to prevent transient ischemic attacks, myocardial infarctions, and artificial heart thrombi formation after valve surgery. Low-dose aspirin can effectively reduce the incidence and mortality of colorectal cancer (132). In this regard, low-dose aspirin taken every other day has been reported to effectively reduce the risk of colorectal cancer in healthy women (133, 134). A study where activated platelets and PANC-1 cancer cells were co-cultivated demonstrated that the proliferation ability was increased by upregulating the expression of c-MYC. After treatment with aspirin, the proliferation rate of cancer cells was significantly reduced, and the expression of c-MYC was suppressed (72). Clopidogrel exhibits a similar antiplatelet effect as aspirin by inhibiting ADP receptors on the surface of platelets. Using an orthotopic PC mouse model, Mezouar et al. (135) revealed that clopidogrel could directly inhibit platelet activation, significantly reducing thrombus formation, tumor growth, and metastasis without increasing the risk of bleeding. Additionally, studies have shown that the activation of EMT progression in PC cells further promoted chemotherapy resistance of PDAC (136). Low molecular weight heparin (LMWH) can reduce direct contact and interaction between platelets and tumor cells through the action of antithrombin, thereby reducing EMT progression induced by platelets (55). However, the risk of treatment-related bleeding is greatly increased by the long-term use of LMWH. Receptor tyrosine kinase (RTK) represents the largest class of enzyme-linked receptors, acting as a receptor and an enzyme that can bind to ligands and phosphorylate tyrosine residues of the target protein. It consists of a ligand-binding site in the extracellular domain, the single-pass hydrophobic α helix region and an intracellular domain with tyrosine-protein kinase (PTK) activity (137). RTK mediates the connection between cells and controls a wide range of complex biological functions, including cell growth, movement, differentiation, and metabolism. Therefore, the dysregulation of the RTK signal leads to various human diseases, including cancer (138).

It has been established that PDGFRs and VEGFRs belong to the RTK supergene family, are widely distributed in the membrane of PC cells and vascular endothelial cells in the TME, and interact with abundant PDGF and VEGF released by platelets. Many experiments have been designed to explore the roles of PDGFR or/and VEGFR in PC tumorigenesis. Current evidence suggests that platelet-derived endothelial cell growth factor harbors angiogenic activity *in vitro* and *in vivo* and contributes to angiogenesis and remodeling in the TME (138). The platelet-derived endothelial cell growth factor is overexpressed in most human cancers and associated with increased microvessel density, tumor aggressiveness and poorer patient prognosis (139). In nude mouse orthotopic tumor model experiments, the phosphorylation of PDGFR in tumor and tumor-associated endothelial cells was found to be significantly inhibited by the administration of GN963, a tyrosine kinase inhibitor against PDGFR and Src kinases. Importantly, the activity of Src and Akt kinases in tumor cells was reduced, resulting in a decrease in microvessel density and cell proliferation and increased apoptosis of tumor and tumor-associated endothelial cells (140).

Pericytes are embedded in the basement membrane of capillary endothelial cells and regulate the proliferation and differentiation of endothelial cells associated with angiogenesis. It has been shown that the surface of pericytes is rich in PDGFR-β (141). The specific inhibition of the PDGFR-β signal eliminates PDGFR-β(+) progenitor perivascular cells and mature pericytes around tumor blood vessels, resulting in excessive expansion of blood vessels, endothelial cell apoptosis and low pericytes coverage in PC (142). Subsequently, eliminating pericytes is conducive to tumor vascular degeneration and significant tumor growth inhibition. Moreover, inhibitors of VEGF signaling can block VEGF-mediated endothelial cell survival, tube formation and downstream signaling, inhibit angiogenesis, and reduce tumor vascular distribution (143, 144), exhibiting no harm to the integrity of blood vessels in normal tissues and organs (145). However, due to the complexity of tumor metabolism in time and space, the efficacy of antitumor drugs is greatly affected. Bergers et al. (146) revealed that SU5416, an inhibitor that targets VEGFR in endothelial cells, was effective for early angiogenic lesions but not for large, well-vascularized tumors in mouse models of PC. In contrast, SU6668, a selective kinase inhibitor of PDGFR, has been shown to prevent further end-stage tumor growth, causing pericyte detachment and tumor blood vessel destruction. The combination of SU5416 and SU6668 was more effective than mono drug therapy at all stages of pancreatic islet carcinogenesis. Other antiplatelet drugs have also been used for the treatment of PC. For example, integrin α-2 is the most expressed integrin molecule on the platelet membrane and mediates platelet adhesion and aggregation. Integrin α-2 inhibitor significantly reduces the microvessel density of PC in mice and effectively inhibits tumor growth (147). In a mice model of PC with liver metastasis, the number of multiple metastatic nodules on the liver surface was significantly reduced after injection of prostaglandin before tumor formation, which might be due to inhibition of platelet aggregation by prostaglandin E1 and I2 (148).

### 5.2 Antiplatelet Combined With Chemotherapy or Radioimmunotherapy in PC

At present, although gemcitabine is still the standard first-line treatment for patients with advanced PC, the benefits of these drugs for the survival of patients with PC are below expectations (136), which may be accounted for by activated platelets weakening the therapeutic effect of antitumor drugs. Platelets interact with PC cells and stimulate the PI3K/Akt and MEK/Erk signaling by releasing activated platelet-derived TGF-β1, causing cancer cell tolerance to cisplatin (114). The activation of platelets in the TME account for increased chemotherapy resistance of pancreatic ductal adenocarcinoma. In an orthotopic PC model in nude mice, the combination of VEGF receptor antibody and gemcitabine inhibited primary pancreatic tumor growth and the incidence of lymphatic metastasis and liver metastasis to a great extent compared to monotherapy, improving the survival rate of mice (149, 150). PKI 166, an EGFR protein tyrosine kinase inhibitor, combined with gemcitabine, could effectively reduce microvessel density, inhibit cell proliferation, increase tumor cell and endothelial cell apoptosis, and significantly inhibit lymph node and liver metastasis (151). Ticagrelor, which inhibits platelet activation through the ADP-P2Y12 axis, can significantly reduce the proliferation ability of PC cells but not normal pancreatic cells. The combination of ticagrelor and gemcitabine significantly has been found to reduce tumor growth *in vivo* (83). In addition, gemcitabine combined with anticoagulants (e.g., dalteparin) can significantly reduce the incidence of vascular thromboembolism in advanced PC and reduce mortality due to vascular thromboembolism (152, 153). Importantly, it has been shown that radioimmunotherapy combined with imatinib (a potent inhibitor of PDGF-β) significantly inhibits the growth of PC compared to radioimmunotherapy alone and does not produce any obvious side effects (154). Experimental studies have shown that SU6668 could increase the radiosensitivity of tumor blood vessels, which contributed to tumor growth inhibition and enhanced tumor response to radiotherapy (155). Adjuvant chemoradiotherapy has been established to play a minimal role in controlling advanced PC and does not improve patient prognosis (156). It remains unknown whether antiplatelet therapy combined with chemoradiotherapy will benefit patients. Overall, antiplatelet therapy can inhibit platelet-related cancer-promoting pathways, and combinations of chemotherapy and radioimmunotherapy can effectively enhance antitumor efficacy (157).

### 5.3 Platelet-Nanotechnology Treatment in PC

The barrier function of tumor vascular endothelial cells is strengthened by adhesion of the covering activated platelets, limiting penetration of chemotherapeutic drugs in the tumor cell yielding a poor antitumor effect. To overcome this problem, Cao et al. (158) constructed TM33 peptide-modified gelatin/oleic acid nanoparticles loaded with TNA that could specifically bind to P-selectin on the surface of activated platelets and release the target drug TNA into the extracellular space under the stimulation of MMP-2 secreted by activated platelets, to induce high local TNA exposure. Platelet activation was inhibited by the high concentrations of TNT on the surface of tumor blood vessels, which improved blood vessel penetration and allowed antitumor chemotherapy drugs to leak into tumor cells. Most importantly, TNT did not cause additional side effects, such as bleeding, without changing the biological functions of platelets. In addition, a small dose of nanoparticle-antitumor drugs coated with platelet membrane could selectively adhere to cells in the TME and improve the bioavailability of the antitumor drug after local delivery. Side effects of systemic high-dose administration, including rapid white blood cell consumption and temporary local immune insufficiency, were not observed (159). Geng et al. (160) constructed a platelet camouflage nanoprobe with active targeting properties, which could escape macrophage phagocytosis and specifically bind to CD44 on the surface of most cancer cells, showing great potential for accurate diagnosis and effective treatment of cancer. Accordingly, platelet-related nanotechnology treatments can reduce the damage caused by drugs to vital human organs and accurately target PC lesions. Nano-combined targeted drug technology has great potential in the treatment of PC.

Although antiplatelet drugs combined with chemotherapeutics effectively improve the antitumor effect, the adverse events caused by these drugs should not be ignored. Common adverse events encompass fatigue, anorexia, dysphonia, nausea and decreased platelet count (161), while serious adverse events include peptic ulcer disease and gastrointestinal bleeding (133, 162). More importantly, it remains controversial whether antiplatelet drugs combined with chemotherapeutics will bring survival benefits compared with chemotherapeutics alone in advanced PC patients (161), raising awareness on the need to develop precise and individualized treatments for this patient population.

### 5.4 Platelet-Related Therapy and PC Organoid

Organoids are three-dimensional (3D) cell cultures that contain key properties of the organs they represent. These *in vitro* culture systems include self-renewing stem cells that can differentiate into multiple organ-specific cell types, exhibiting a similar spatial organization to their counterparts and reproducing some of their functions. Accordingly, they can mimic human development. and disease potential, thus providing a physiologically relevant system (163). It has been shown that PC organoids can be rapidly generated from resected human or mouse tumors and biopsies with success rates as high as 75-83%. A comprehensive transcriptional and proteomic analysis of pancreatic organoids could reveal key genes and pathways altered during disease progression (164). Given that 85% of PC patients are not indicated for surgery (165), PC organoids can be generated from limited amounts of cellular material provided by endoscopic ultrasound-guided fine-needle aspiration (EUS-FNA) to detect differences in gene profile expression and find diagnostic and personalized treatment approaches (164). Moroever, a comprehensive genomic, transcriptomic and therapeutic analysis of PC patient-derived organoids (PDOs) could identify molecular and functional subtypes of PC, predict treatment response, and facilitate precision medicine for this patient population (166). Interestingly, establishing a platelet co-culture model with PC organoid can simulate the crosstalk between PC, extracellular matrix and platelets in the TME and reveal the underlying mechanisms of the interaction between PC cells and platelets (167). However, exploring the mechanisms of PC development through platelets (platelet-related therapy) and organoid co-culture models is at an early stage, warranting more experimental data to substantiate current findings.

## 6 Conclusion

In conclusion, unprecedented progress has been made in better understanding platelet-mediated signaling pathways in recent years. Platelet-based studies provide novel insights into how platelets work and the basis to develop targeted therapies that can improve patient outcomes. Although the mechanisms of PC cells in escaping NK cells to lead to distant metastasis have been understood, to some extent, it remains unclear whether PC cells escape other immune cells *via* the same mechanisms. Improving immune cell monitoring and killing ability against cancer cells may be a potential approach for PC treatment. Importantly, antiplatelet therapy combined with radiotherapy or chemotherapy and platelet-related nanotechnology *in vitro* and in animal models are being investigated in ongoing studies, and the clinical efficacy has not been evaluated. Platelets and PC organoid co-culture models have important application value in discovering key points in platelet-induced PC pathogenesis and treatment. Furthermore, given the high incidence of peptic ulcers and gastrointestinal bleeding caused by antiplatelet drugs, scientific and reasonable approaches should be emphasized to ensure patient safety. A better understanding of platelet-mediated signaling pathways will provide a solid foundation for improving patient care. Indeed, comprehensive methods combining immunotherapy, chemotherapy and nanotechnology can potentially benefit PC patients.

## Author Contributions

ZC, XW, and WZ conceived the review. ZC, XW, SD, FH and RH undertook the initial research. ZC, XW, SD, FH, and RH were involved in writing. ZC reviewed the manuscript, and all authors contributed to the final version. XW contributed equally to this work and should be considered co-first author. All authors contributed to the article and approved the submitted version.

## Funding

This article was supported by The First Hospital of Lanzhou University Intra-Hospital Fund Youth Fund, ldyyn2020-76.

## Conflict of Interest

The authors declare that the research was conducted in the absence of any commercial or financial relationships that could be construed as a potential conflict of interest.

## Publisher’s Note

All claims expressed in this article are solely those of the authors and do not necessarily represent those of their affiliated organizations, or those of the publisher, the editors and the reviewers. Any product that may be evaluated in this article, or claim that may be made by its manufacturer, is not guaranteed or endorsed by the publisher.
